# Incidence of Respiratory Symptoms for Residents Living Near a Petrochemical Industrial Complex: A Meta-Analysis

**DOI:** 10.3390/ijerph17072474

**Published:** 2020-04-04

**Authors:** Wen-Wen Chang, Hathaichon Boonhat, Ro-Ting Lin

**Affiliations:** 1 Department of Occupational Safety and Health, College of Public Health, China Medical University, Taichung 40402, Taiwan; u106026468@cmu.edu.tw; 2 Graduate Institute of Public Health, College of Public Health, China Medical University, Taichung 40402, Taiwan; u107050121@cmu.edu.tw

**Keywords:** respiratory symptoms, petrochemical industrial complexes, air pollution, residential exposure, meta-analysis

## Abstract

The air pollution emitted by petrochemical industrial complexes (PICs) may affect the respiratory health of surrounding residents. Previous meta-analyses have indicated a higher risk of lung cancer mortality and incidence among residents near a PIC. Therefore, in this study, a meta-analysis was conducted to estimate the degree to which PIC exposure increases the risk of the development of nonmalignant respiratory symptoms among residents. We followed the Preferred Reporting Items for Systematic Reviews and Meta-Analyses (PRISMA) guidelines to systematically identify, select, and critically appraise relevant research. Finally, we identified 16 study groups reporting 5 types of respiratory symptoms: asthma, bronchitis, cough, rhinitis, and wheezing. We estimated pooled odds ratios (ORs) using random-effect models and investigated the robustness of pooled estimates in subgroup analyses by location, observation period, and age group. We determined that residential exposure to a PIC was significantly associated with a higher incidence of cough (OR = 1.35), wheezing (OR = 1.28), bronchitis (OR = 1.26), rhinitis (OR = 1.17), and asthma (OR = 1.15), although the latter two associations did not reach statistical significance. Subgroup analyses suggested that the association remained robust across different groups for cough and bronchitis. We identified high heterogeneity for asthma, rhinitis, and wheezing, which could be due to higher ORs in South America. Our meta-analysis indicates that residential exposure to a PIC is associated with an increased risk of nonmalignant respiratory symptoms.

## 1. Introduction

The petrochemical industry is frequently a large contributor to a country’s economic development and is a source of employment opportunities for communities [1]. However, the health of those residing near petrochemical industrial complexes (PICs) is a critical concern due to potential pollution emissions. Industrial plants can increase the concentration of hazardous substances in surrounding areas and cause respiratory symptoms among residents [2,3]. The operations of PICs produce various air pollutants, such as volatile organic compounds, sulfur dioxide, particulate matter, and nitrogen oxides [4,5,6,7,8]. Exposure to these pollutants is associated with incidence of asthma, cough, wheezing, and bronchitis [9,10,11,12]. 

Residential exposure to PICs is significantly associated with various respiratory symptoms [7,8,12,13,14]. For example, one study in Spain used the International Study on Asthma and Allergies in Children questionnaire to survey the population and observed a higher prevalence of nocturnal cough among children aged 6–7 years and adolescents aged 13–14 years who lived within 4.7 of a PIC in Tarragona compared with those living in areas of the country without a PIC [14]. In South America, studies have identified associations between residential proximity to a PIC and asthma, rhinitis, cough, and wheezing [8,13]. A study in Brazil revealed that residents aged 0–14 living within a 5-km radius of a PIC had an increased prevalence of wheezing; male residents were twice as likely as female residents to have wheezing [13]. In addition, a study in Argentina determined that children living near PICs had an increased risk of asthma, wheezing, nocturnal cough, and allergic rhinitis; the length of residence near the polluted area was a significant risk factor [8]. In Asia, similar associations between residential PIC exposure and respiratory symptoms were observed among young residents (aged 6–13 years) in Taiwan and also among older residents (aged > 40 years) in Thailand [7,12]. A study in Thailand determined that residents living within a 10-km radius of the Map Ta Phut Industrial Estate aged > 40 had an increased risk of chronic cough, bronchitis, wheezing, and lower respiratory tract symptoms lasting at least 3 months; prolonged residence near the PIC increased the risk of wheezing and upper respiratory symptoms [7]. A study in Taiwan identified higher levels of sulfur dioxide, particulate matter, and nitrogen dioxides surrounding PICs and revealed that nearby residents had a significantly greater risk of experiencing asthma and upper respiratory symptoms [12].

By contrast, studies elsewhere have not demonstrated significant associations between residential proximity to a PIC and the aforementioned respiratory symptoms [15,16]. A study in the United Kingdom revealed no significant association between residential proximity to a PIC and asthma or bronchitis [15]. Additionally, in a study in Changhua, Taiwan, a standardized questionnaire was used to survey participants on the prevalence of allergic respiratory diseases and symptoms; no significant association was identified between living in Taihao, which had severe air pollution due to a PIC, and prevalence of wheezing, asthma, or rhinitis [16].

Two meta-analyses investigated the association between PIC exposure and lung cancer mortality and incidence, respectively [17,18]. However, neither pooled data analysis nor a meta-analysis have reported the impact of PICs on nonmalignant respiratory symptoms. Therefore, the aim of the present study was to conduct a meta-analysis to estimate the degree to which PIC exposure is associated with the development of nonmalignant respiratory symptoms among nearby residents.

## 2. Materials and Methods 

### 2.1. Search Strategy

We followed the Preferred Reporting Items for Systematic Reviews and Meta-Analyses (PRISMA) guidelines to search literature and select studies. We defined “(refinery OR petroleum OR petrochemical OR oil and gas industry) AND ((respiratory symptom) OR (signs and symptoms, respiratory))” as our search terms. Two authors (W.W.C. and H.B.) separately searched for studies available in the period January 1, 1971 to January 9, 2020 in PubMed, Web of Science, EBSCOhost, Cochrane Library, ClinicalKey, and Embase databases.

### 2.2. Study Eligibility Criteria and Selection

**Figure 1:**
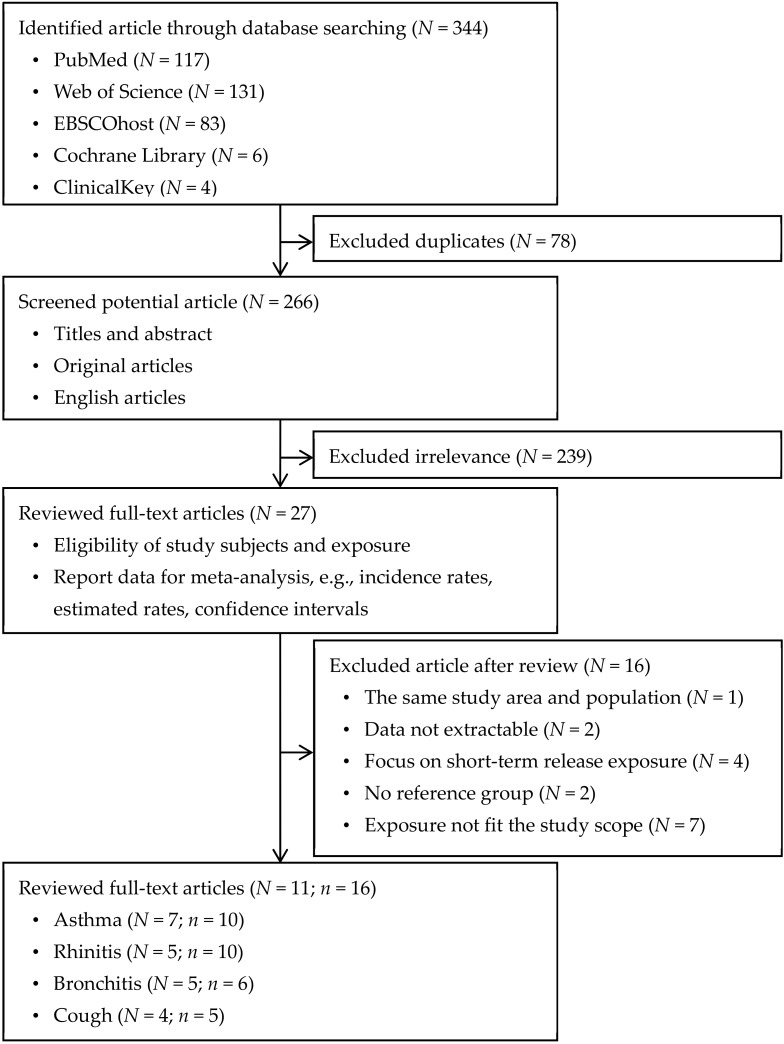
Search process for reports on respiratory symptoms among residents living near petrochemical industrial complexes. Abbreviations: *N*, number of articles; *n*, number of study groups included in the meta-analysis.

Figure 1 illustrates the study identification, screening, review, and selection procedures. We identified 344 articles from the 6 databases in the initial step. In the second step, we excluded 78 duplicates and then screened 239 potential articles. After screening, we included 27 relevant articles according to the following criteria: the title and abstract focused on the respiratory health of residents near a PIC, and the full-text articles were available in English. In the third step, we reviewed these 27 articles and further subjected them to the following selection criteria: (1) the studies included defined exposure and reference groups, (2) the exposure group was defined as residents near a PIC, (3) the studies were not conducted on the same study area or population as other studies, (4) the studies employed an area-based approach, and (5) the studies had computable data. We excluded 16 studies because they did not include reference groups, their study areas and populations overlapped, they focused on short-term exposure, or they involved unusable estimates. Finally, 11 studies were retained for meta-analysis.

### 2.3. Data Extraction and Analysis

These studies reported eight types of nonmalignant respiratory symptoms. After the exclusion of three types of symptoms within ≤2 study groups [8,14], our meta-analysis finally included five types of nonmalignant respiratory symptoms: asthma, bronchitis, cough, rhinitis, and wheezing. Table 1 presents the definitions of the five symptoms. According to these criteria and definitions, 16 study groups were identified from the 10 articles for meta-analysis. Regarding types of symptoms, 5 groups from 4 studies reported cough, 6 groups from 5 articles reported bronchitis, 10 groups from 5 articles reported rhinitis, 10 study groups from 7 articles reported asthma, and 10 groups from 7 studies reported wheezing.

We obtained prevalence, adjusted odds ratios (ORs), and 95% confidence intervals (CIs) for the 16 study groups. We organized data according to the five aforementioned respiratory symptoms and applied random-effect models to analyze the pooled risk of the respiratory symptoms in residents near PICs, which accounted for between-study variation of heterogeneity [24,25]. This model allows for heterogeneity of pooled risk by assuming that the effects are normally distributed. The I^2^ test was applied to address heterogeneity between the collected study groups, with 10% indicating no heterogeneity, 10–30% indicating low heterogeneity, 30–60% indicating moderate heterogeneity, and >60% indicating high heterogeneity [26]. We used funnel plots to assess publication bias and conducted statistical testing using Egger’s test [27].

We performed subgroup analyses to examine the robustness of pooled estimates using different locations, observation periods, and age groups of the study populations. According to the median of observation periods reported in the 11 studies, we divided observation periods into two categories: ≥3 and <3 years. We reviewed population age in each study and defined the following age subgroups: 6–8 years representing the “junior group”, 11–14 years representing the “intermediate group”, and 0–14 years (or those whose ages are not known) representing the “wide group”. Finally, we applied a funnel plot to examine whether publication bias was present across studies.

### 2.4. Study Quality

To assess the quality of the studies and incorporate quality assessment into our interpretation of the results, we used the Newcastle-Ottawa Quality Assessment Scale adapted for cross-sectional studies [28]. We evaluated potential bias in selection, comparability, and outcome [28]. We assigned stars to evaluate study quality, with nine to ten stars indicating “very good” quality, seven to eight stars indicating “good” quality, five to six stars indicating “satisfactory” quality, and zero to four stars indicating “unsatisfactory” quality [29].

Table S1 lists the results of the quality assessment for all cross-sectional studies. One study (nine stars) was very good [30]. Eight studies (six with eight stars and two with seven) were good [8,12,13,14,15,16,31,32]. Two studies (six stars) were satisfactory [7,33].

### 2.5. Trial Registration

Our study was registered on PROSPERO on 26 September 2019. The registration number is 152438.

## 3. Results

Appendix A Table A1 presents the characteristics of the study groups included in the meta-analysis. The collected studies included approximately 14,532 residents who might be exposed to PICs in seven countries: Argentina (La Plata), Brazil (Rio Grande do Norte), Taiwan (Miaoli, Jenwu, Linyuan, Chunghua, and Yunlin), Thailand (Rayong Province), Spain (Tarragona), Italy (Sardinia and Basilicata), and the United Kingdom (Teesside). The study period ranged from 1981 to 2017.

**Figure 2:**
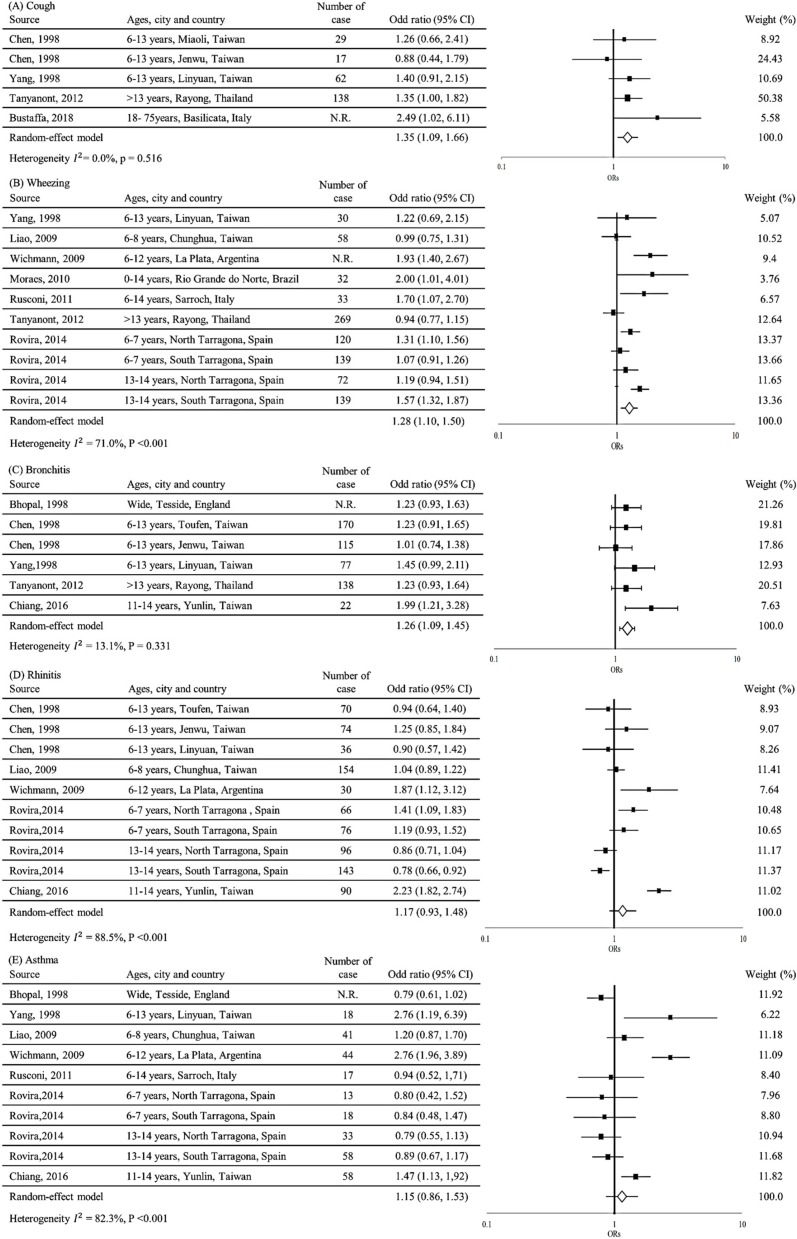
Forest plots of studies on five respiratory symptom risks among residents near petrochemical industrial complexes. (**A**) Forest plot of studies on cough; (**B**) forest plot of studies on wheezing; (**C**) forest plot of studies on bronchitis; (**D**) forest plot of studies on rhinitis; (**E**) forest plot of studies on asthma. N.R., nonreported; 95% CIs, 95% confidence intervals; Weight, the estimate for the study accounted for the weight proportion.

**Figure 3:**
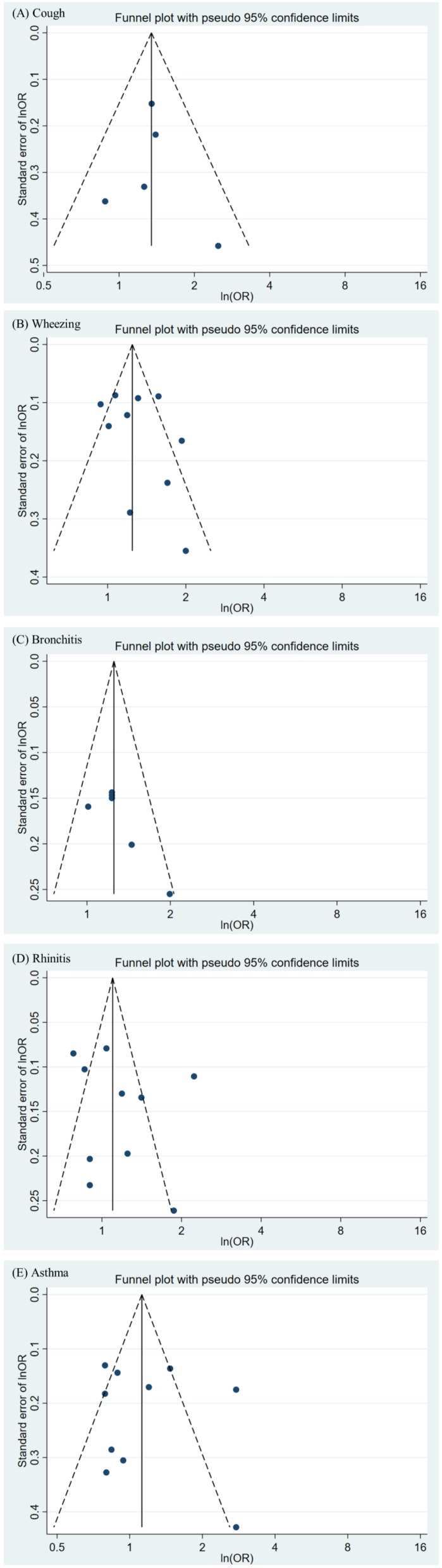
Funnel plot of study groups and risk of five respiratory symptoms for residents near petrochemical industrial complexes. (**A**) Funnel plot of study groups on cough; (**B**) funnel plot of study groups on wheezing; (**C**) funnel plot of study groups on bronchitis; (**D**) funnel plot of study groups on rhinitis; (**E**) funnel plot of study groups on asthma. N.R., nonreported; 95% CIs, 95% confidence intervals.

Figure 2 details the pooled estimates of the risk for the five respiratory symptoms among the residents. As illustrated in Figure 2A, an overall pooled OR of 1.35 (95% CI = 1.09−1.66) indicated that incidence of cough among residents was significantly associated with exposure to a PIC. The overall I^2^ was 0.0%, indicating no heterogeneity for cough in our meta-analysis. Due to its narrow 95% CI (at 1.00–1.82), the estimate of a study in Thailand accounted for the largest percentage weight (50.38%). The estimates for cough across the four study groups had no asymmetry (Egger’s test, *p* value = 0.868) (Figure 3A).

As presented in Figure 2B, an overall pooled OR of 1.28 (95% CI = 1.10–1.50) indicated that the incidence of wheezing among residents was significantly associated with exposure to a PIC, despite two of the ten study groups reporting point estimates of <1. The overall I^2^ was 71.0%, signifying high heterogeneity for wheezing in our meta-analysis. We observed that all Asian study populations had non-significant pooled estimates, whereas all study populations from the Americas had significant pooled estimates. Although the data collected from a Brazilian study had an OR of 2.00, the estimate for this study accounted for only 3.76% of the weight proportion because of its wide 95% CI (1.01–4.01). Publication bias was observed for wheezing among ten study groups because three were outside the confidence limits. Despite being outside the limits, two of the three study groups were from studies that had more than seven stars (i.e., from good studies); the other study group (from a satisfactory study) was closer to the confidence limits (Egger’s test, *p* value = 0.393) (Figure 3B). 

As displayed in Figure 2C, an overall pooled OR of 1.26 (95% CI = 1.09–1.45) indicated that bronchitis incidence among residents was significantly associated with exposure to a PIC. The overall I^2^ was 13.1%, signifying low heterogeneity for bronchitis in our meta-analysis. All studies had pooled estimates greater than 1. The highest OR was observed for Taiwan at 1.99, but it accounted for only 7.63% of the proportion weight because of its wide 95% CI (at 1.21–3.28). The estimates for bronchitis among the six study groups had no asymmetry (Egger’s test, *p* value = 0.054) (Figure 3C).

As displayed in Figure 2D, the estimated overall OR of 1.17 signified that rhinitis incidence among residents may be associated with exposure to a PIC, although it did not reach statistical significance (95% CI = 0.93–1.48); nevertheless, four in ten studies reported pooled estimates lower than 1. The overall I^2^ was 88.5%, implying high heterogeneity for rhinitis in our meta-analysis. We observed that a European study population (aged 13–14 years) had pooled estimates that were less than 1. As detailed in Figure 3D, three study groups were outside the confidence limits. Despite the high publication bias, these three groups were from studies that had more than seven stars (Egger’s test, *p* value = 0.479).

As shown in Figure 2E, an overall pooled OR of 1.15 (95% CI = 0.86–1.53) indicated that asthma incidence was non-significantly associated with exposure to a PIC. All European study populations (*n* = 6) had pooled estimates of less than 1, whereas the study populations of other regions (*n* = 4) had pooled estimates greater than 1. Furthermore, the study conducted in Taiwan had the highest OR (2.76), but it had the lowest weighting factor of 6.22% because of its wider 95% CI (i.e., 1.19–6.39). The overall I^2^ was 82.4%, indicating high heterogeneity for asthma in our meta-analysis. As may be observed in Figure 3E, three study groups were outside the confidence limits for publication bias. Despite being outside the limits, the groups were from studies having more than seven stars (Egger’s test, *p* value = 0.767).

**Figure 4:**
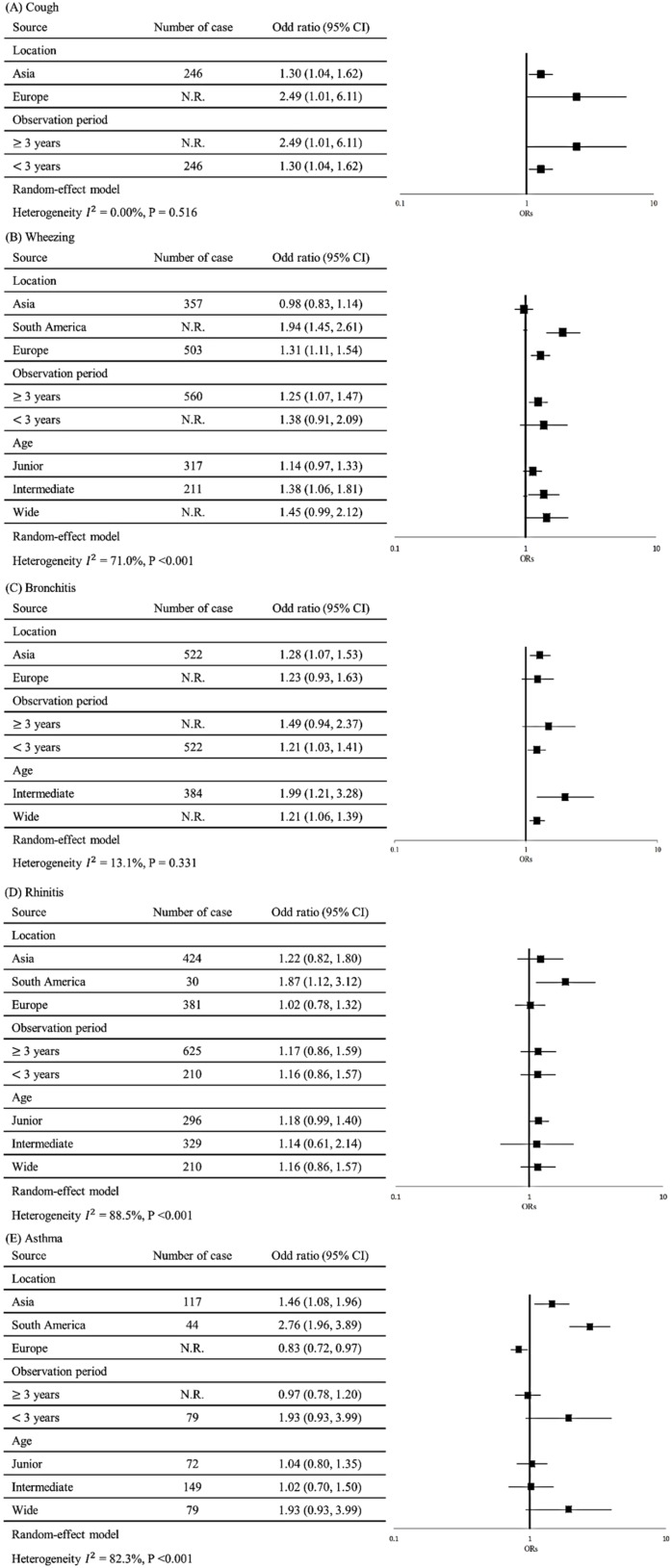
Subgroup analyses of studies on the risk of five respiratory symptoms among residents near petrochemical industrial complexes. (**A**) Subgroup analyses of studies on cough; (**B**) subgroup analyses of studies on wheezing; (**C**) subgroup analyses of studies on bronchitis; (**D**) subgroup analyses of studies on rhinitis; (**E**) subgroup analyses of studies on asthma. N.R., nonreported; 95% CIs, 95% confidence intervals.

Figure 4 details the results of our subgroup analysis for five respiratory diseases or symptoms among residents near a PIC. Results stratified by observation period indicated that the longer the observation period was, the easier it became to observe cough symptoms (Figure 4A). Results stratified by location indicated that residents near PICs in the Americas were at a higher risk of wheezing (OR = 1.94; 95% CI = 1.45–2.61) compared with those in Europe (OR = 1.31; 95% CI = 1.11–1.54) and Asia (OR = 0.98; 95% CI = 0.83–1.14) (Figure 4B). After stratification by observation period, we determined that the longer the observation period was, the easier it became to observe wheezing symptoms. After stratifying by age, we noted that those in the wide study population were at a higher risk of wheezing (OR = 1.45; 95% CI = 0.99–2.12) compared with those aged 11–14 years (OR = 1.38; 95% CI = 1.06–1.81) and aged 6–8 years (OR = 1.14; 95% CI = 0.97–1.33).

As presented in Figure 4C, the results stratified by location indicated that residents near a PIC in Asia were at a higher risk of bronchitis (OR = 1.28; 95% CI = 1.07–1.53) compared with those in Europe (OR = 1.23; 95% CI = 0.93–1.63). After stratification by observation period, we observed that the pooled OR derived for the observation period of ≥3 years was 1.49 (95% CI = 0.94–2.37) relative to that for the observation period of <3 years (OR = 1.20; 95% CI = 1.03–1.41). The highest OR of 1.99 (95% CI = 1.21–3.28) was observed for a Taiwanese study that was conducted over 12 years. It accounted for only 7.63% of the weight proportion because of its wide 95% CI (1.21–3.28). After stratification by age, we noted that those aged 11–14 years were at a higher risk of bronchitis (OR = 1.99; 95% CI = 1.21–3.28) relative to the wider study population (OR = 1.21; 95% CI = 1.06–1.39).

As displayed in Figure 4D, the results stratified by location indicated that the residents near a PIC in the Americas were at higher risk of rhinitis (OR = 1.87; 95% CI = 1.12–3.12) compared with those in Asia (OR = 1.22; 95% CI = 0.82–1.80) and Europe (OR = 1.02; 95% CI = 0.78–1.32). After stratification by age, we determined that although the three age groups were all non-significant, those aged 6–8 years had a higher risk of rhinitis (OR = 1.18; 95% CI = 0.99–1.40) compared with those in the wider study group (OR = 1.16; 95% CI = 0.86–1.57) and those aged 11–14 years (OR = 1.14; 95% CI = 0.61–2.14).

As presented in Figure 4E, the results stratified by location suggested that residents near a PIC in the Americas were at a higher risk of asthma (OR = 2.76; 95% CI = 1.96–3.89) compared with those in Asia (OR = 1.46; 95% CI = 1.08–1.96) and Europe (OR = 0.83; 95% CI = 0.72–0.97). After stratification by age, although the estimates of the three age groups were all non-significant, those in the wide study population were at higher risk of asthma (OR = 1.93; 95% CI = 0.93–3.99) compared with those aged 6–8 years (OR = 1.04; 95% CI = 0.80–1.35) and those aged 11–14 years (OR = 1.02; 95% CI = 0.70–1.50).

## 4. Discussion

Environmental pollutants from PIC operations have been associated with an increased prevalence of respiratory symptoms [8,12,14]. By integrating data from previous studies that associated diverse respiratory symptoms with PICs, our meta-analysis revealed a higher incidence of cough (OR = 1.35; 95% CI = 1.09–1.66), wheezing (OR = 1.28; 95% CI = 1.10–1.50), bronchitis (OR = 1.26; 95% CI = 1.09–1.45), rhinitis (OR = 1.17; 95% CI = 0.93–1.48), and asthma (OR = 1.15; 95% CI = 0.86–1.53) among residents near PICs, although the latter two associations did not reach statistical significance.

The order of the risk of the five respiratory symptoms approximated the order in which particles and chemical toxicants enter the respiratory tract and cause inflammation or other symptoms. Cough is a basic symptom of respiratory disease [23]. When chemical irritants enter the airway, they may be caught by airway mucus and removed by cilia [23]. Wheezing is also a basic symptom of respiratory disease [23]. When irritants enter the airway, widespread smooth muscle contraction may occur and mucus secretion become excessive. These responses narrow and obstruct the airway to cause wheezing [23]. The onset of rhinitis is in the upper respiratory tract, and the onsets of asthma and bronchitis are in the lower respiratory tract [34,35]. When pollutants such as PM_10_ enter the respiratory tract, they may be stopped at the nasal cavity, thus causing rhinitis. Fine particles can enter further into the tracheobronchial tree (thus causing asthma), travel to the bronchioles and alveolar parts of the lung, and even enter the bloodstream [36,37]. Pollutants can be sensed first by airway epithelial cells and then by active macrophages and innate immune cells [38,39]. These responses may cause the release of numerous inflammatory molecules [36,40]. Thus, pollutants typically cause rhinitis first and then asthma attack and bronchitis. Additionally, inflammatory molecules stimulate surrounding tissues and cause bronchitis [41].

Our subgroup analyses revealed that some groups bear a higher risk, namely those in studies conducted in South America or in studies with a longer observation period. This first result is comparable to the incidence rate estimated by the Global Burden of Diseases, Injuries, and Risk Factors Study [42]. Taking asthma incidence as an example, the rate of people in Latin America and the Caribbean (855.28 per 100,000 people) was higher than of those in Asia (482.79 per 100,000 people) and Europe (399.89 per 100,000 people) [42]. Regarding observation periods, we divided it into two categories at 3 years, in contrast to a previous study that divided the period into 20 plus 10 years [17]. This is because the previous study focused on cancer, which requires researchers to consider the latency period [17]. Nonetheless, their study and ours are consistent in observing that a longer observation period may result in the detection of a higher risk of respiratory symptoms [17].

However, our study has some limitations. First, the included articles compared risk based on areas or geographic regions as defined by the authors rather than on absolute distance to PICs. We may underestimate the risk of respiratory symptoms for people living closer to PICs. Future studies using distance as a continuous variable might provide a more accurate estimation. Second, potential confounders might not have been comprehensively considered and adjusted for in some of the original studies. For example, smoking was not adjusted for in all the studies. Although smoking is less prevalent among residents in counties with a refinery (54%) than in counties without one (70%) [43], we may have underestimated differences between the risks resulting from exposure to PICs and reference groups. Additionally, PIC operations in different countries may emit variable types of air pollutants [44]. Amounts of air pollutants influence many types of respiratory symptoms [9,10,11,12]. A separate study measuring the levels of pollutants around PICs in various countries is suggested. A third limitation is its implications for other countries. The study populations covered residents near PICs in only seven countries. Thus, caution should be exercised when extrapolating our findings to other countries. A fourth limitation is the inconsistency of pollutants in the included papers. Moreover, the contribution of each pollutant to impairments of respiratory health remains unclear. Typically, a high concentration of one pollutant is often related to a high concentration of other pollutants [12]. Exposure to a complex of pollutants may be associated with multiple respiratory symptoms. This topic is beyond the scope of this meta-analysis; hence, a future study should directly quantify the health effect of multiple pollutants [45]. Finally, in our study, a meta-analysis was conducted to estimate the degree to which PIC exposure affects the health of residents and did not focus on workers. A study of its impact on workers may be warranted [46].

## 5. Conclusions

The size of the global petrochemicals market is increasing. Our meta-analysis confirmed significant associations between residential proximity to PICs and the risk of nonmalignant respiratory symptoms. The order of the risk of five respiratory symptoms approximate the order in which particles and chemical toxicants enter the respiratory tract and cause inflammation or other symptoms. Our findings suggest additional in-depth studies are required to explore other reasons behind high respiratory risks among people in South America as well as to document and quantify the effect of each pollutant on impairments of respiratory health.

## Figures and Tables

**Table 1:** Definitions of five nonmalignant respiratory symptoms.

Respiratory Symptoms	Definition	Source
Cough	It’s a mechanism that lung airways clear fluids, mucus, or other material. It may typically be the result of inhaling foreign objects into the lung.	[19]
Bronchitis	It’s an infection of the main airway of the lungs, causing them to become irritated and inflamed. Individuals with bronchitis have a reduced ability to breathe; also, they have sore throat, wheezing and cough.	[20]
Rhinitis	It’s inflammation of the inside of the nose caused by an allergen. The inside of nose becomes inflamed and swollen.	[21]
Asthma	Chronic disease of the airways which is due to inflammation of the air passages in the lungs, affects the sensitivity of the nerve endings in the airways and swells.	[22]
Wheezing	It’s a high-pitched whistling sound made while you breathe. It’s caused by narrowed airways or inflammation.	[23]

## Supplementary Materials

The following are available online at https://www.mdpi.com/1660-4601/17/7/2474/s1, Table S1: Newcastle-Ottawa Quality Assessment Scale adapted for Meta-Analysis.

## Appendix A

**Table A1:** Basic characteristics of the studies included in the meta-analysis.

Source	Study Period	Outcome Selection	Adjusted Confounders	Country (City)	Pollutants	Total Number of Exposure	Total Number of Non-Exposure	Age of Population	Case of Exposed Disease	Odd Ratio
Bhopal, 1998 [15]	1981–1994	Adjusted odds ratio (bronchitis, asthma)	Age, sex, smoking, alcohol consumption, exercise level, occupation in heavy industry, damp housing, coal fires, and gas appliances	England (Teesside)	NO_2_, SO_2_	1539	1910	Wide	See note ^1^	Bronchitis: 1.23 (0.93, 1.63) Asthma: 0.79 (0.61, 1.02)
Chen, 1998 [32]	1994–1995	Adjusted odds ratio, prevalence (cough, bronchitis, rhinitis)	Sex, grade, father’s education, crowding index, household smoking, pets, fowls, coal stove used, gas-cooker used, incense burning the whole day, mosquito repellent burning, and home dampness	Taiwan (Miaoli)	Non-methane hydrocarbons (NMHCs), NO_x,_ PM_10_, SO_2_, THC	748	611	6–13 years	Cough: 29 cases Bronchitis: 170 cases Rhinitis: 70 cases	Cough: 1.26 (0.66, 2.41) Bronchitis: 1.23 (0.91, 1.65) Rhinitis: 0.94 (0.64, 1.40)
Chen, 1998 [32]	1994–1995	Adjusted odds ratio, prevalence (cough, bronchitis, rhinitis)		Taiwan (Jenwu, Kaohsiung)	CO, ozone (O_3_), NMHCs, NO_x,_ PM_10_, SO_2_, THC	675	611		Cough: 17 cases Bronchitis: 115 cases Rhinitis: 74 cases	Cough: 0.88 (0.44, 1.79) Bronchitis: 1.01 (0.74, 1.38) Rhinitis: 1.25 (0.85, 1.84)
Chen, 1998 [32]	1994–1995	Adjusted odds ratio, prevalence (rhinitis)		Taiwan (Linyuan, Kaohsiung)	CO, ozone (O_3_), NMHCs, NO_x_, PM_10_, SO_2_, THC	470	611		Rhinitis: 36 cases	Rhinitis: 0.90 (0.57, 1.42)
Yang, 1998 [12]	1994–1995	Adjusted odds ratio, prevalence (cough, wheezing, bronchitis, asthma)	Sex, age, parent education, household smokers, age of home >10 years, pets, carpets, incense burning, gas cooking at home, mosquito repellent burning, plants inside home, and home dampness	Taiwan (Linyuan)	NO_2_, PM_10_, SO_2_, acid aerosols (SO_4_^2−^, NH_4_^+^, NO_3_^-^)	470	611	6–13 years	Cough: 62 cases Wheezing: 30 cases Bronchitis: 77 cases Asthma: 18 cases	Cough: 1.40 (0.91, 2.15) Wheezing: 1.22 (0.69, 2.15) Bronchitis: 1.45 (0.99, 2.11) Asthma: 2.76 (1.19, 6.39)
Liao, 2009 [16]	2002, 2007	Prevalence (wheezing, rhinitis, asthma)	Social economic status, environment exposure	Taiwan (Chunghua)	PM_2.5–10_	565	875	6–8 years	Wheezing: 58 cases Rhinitis: 154 cases Asthma: 41 cases	See note^1^
Wichmann, 2009 [8]	2005–2006	Adjusted odds ratio (wheezing, rhinitis, asthma)	Age, sex, environmental tobacco smoke, living near less polluted area, time of residence in study area, home environment, length of exclusive breast-feeding, and family socioeconomic and demographic	Argentina (La Plata)	Particulate matter (PM_0.5–10_), VOC_S_	290	303	6–12 years	Rhinitis: 30 cases Asthma: 44 cases	Wheezing: 1.93 (1.39, 2.67) Rhinitis: 1.87 (1.12, 3.12) Asthma: 2.76 (1.96, 3.89)
Moraes, 2010 [13]	2006–2010	Adjusted odds ratio, prevalence (wheezing)	Age, sex	Brazil (Rio Grande do Norte)	Black carbon, NO_2_, PM_2.5–10_, SO_2_, VOCs	95	114	0–14 years	Wheezing: 32 cases	Wheezing: 2.00 (1.01, 4.01)
Rusconi, 2011 [33]	2006–2007	Prevalence (wheezing, asthma)	Age, sex, parental history of asthma, parent education, passive smoking, damp or mold in child’s bedroom	Italy (Sarroch)	Benzene, NO_2_, O_3_, SO_2_	275	214	6–14 years	Wheezing: 33 cases Asthma: 17 cases	See note^1^
Tanyanont, 2012 [7]	2007–2008	Adjusted odds ratio, prevalence (cough, wheezing, bronchitis)	Age, sex, smoking indoors, environmental exposure within 500m of the residence, length of residence	Thailand (Rayong)	VOCs	8175	7266	>13 years	Cough: 138 cases Wheezing: 269 cases Bronchitis: 138 cases	See note^1^
Rovira, 2014 [14]	2010–2014	Prevalence (wheezing, rhinitis, asthma)	Sex, length of residence, parents’ nationality, parental history of asthma, family affluence scale, and passive and active smoking	Spain (North Tarragona)	VOCs	304	340	6–7 years	Wheezing: 120 cases Rhinitis: 66 cases Asthma: 13 cases	See note^1^
Rovira, 2014 [14]	2010–2014	Prevalence (wheezing, rhinitis, asthma)		Spain (South Tarragona)		401	340		Wheezing: 139 cases Rhinitis: 76 cases Asthma: 18 cases	See note^1^
Rovira, 2014 [14]	2010–2014	Prevalence (wheezing, rhinitis, asthma)	Sex, length of residence, parents’ nationality, parental history of asthma, family affluence scale, and passive and active smoking	Spain (North Tarragona)		269	405	13–14 years	Wheezing: 72 cases Rhinitis: 96 cases Asthma: 33 cases	See note^1^
Rovira, 2014 [14]	2010–2014	Prevalence (wheezing, rhinitis, asthma)		Spain (South Tarragona)		427	405		Wheezing: 139 cases Rhinitis: 143 cases Asthma: 58 cases	See note^1^
Chiang, 2016 [30]	1999–2010	Prevalence (bronchitis, rhinitis, asthma)	Age, sex, living near roads, incense burning, and passive household smoking	Taiwan (Yunlin)	SO_2_	216	371	11–14 years	Bronchitis: 22 cases Rhinitis: 90 cases Asthma: 58 cases	See note^1^
Bustaffa, 2018 [31]	2014–2017	Prevalence (cough)	Age, sex, BMI (body mass index), smoking, cardiovascular comorbidity, occupational exposure, exposure to chemical-physical agents, and distance from the main road.	Italy (Basilicata)	CO, NO_x_, SO_2_, VOCs, NMHCs	91	100	18–75 years	See note^1^	Cough: 2.49 (1.02, 6.11)

^1^ Original articles did not report estimated odds ratio. We collected the number of people who were exposed to PICs and presenting respiratory symptoms, the number of people who were exposed to PICs but not presenting respiratory symptoms, the number of people who were not exposed to PICs but presenting respiratory symptoms, and the number of people who were not exposed to PICs and were not presenting respiratory symptoms. We then used these data to calculate odd ratios.
