# Supplementary material for: Incidence of Respiratory Symptoms for Residents Living Near a Petrochemical Industrial Complex: A Meta-Analysis
Source: Int J Environ Res Public Health. 2020 Apr 4;17(7):2474. doi: 10.3390/ijerph17072474 (PMC7178237; doi:10.3390/ijerph17072474)
Supplement: Supplementary file 1 [file ijerph-17-02474-s001.pdf]

Table S1. Newcastle-Ottawa Quality Assessment Scale adapted for meta-analysis.

		Selection				Comparability	Outcome		
**Source**	**Title**	**Representa-Tiveness of the Sample** [a]	**Sample Size** [b]	**Non-Respondents** [c]	**Ascertainment of the Exposure** [d]	**The Subjects in Different Outcome Groups are Comparable** [e]	**Assessment of the Outcome** [f]	**Statistical Test** [g]	**Quality** [h]
Bhopal, 1998 [15]	Does living near a constellation of petrochemical, steel, and other industries impair health?	*	*	*		**	*		satisfactory
Chen, 1998 [32]	Adverse effect of air pollution on respiratory health of primary school children in Taiwan	*	*			**	*	*	satisfactory
Yang, 1998 [12]	Respiratory symptoms of primary school children living in petrochemical polluted area in Taiwan	*	*	*	*	**	*	*	good
Liao, 2009 [16]	Prevalence of allergic diseases of schoolchildren in center Taiwan: from ISAAC surveys 5 years apart	*	*	*	*	**	*	*	good
Wichmann, 2009 [8]	Increased asthma and respiratory symptoms in children exposed to petrochemical pollution	*	*		**	**	**	*	very good
Moraes, 2010 [13]	Wheezing in children and adolescents living next to a petrochemical plant in Rio Grande do Norte, Brazil	*	*	*	*	**	*	*	good
Rusconi, 2011 [33]	Asthma symptoms, lung function, and makers of oxidative stress and inflammation in children exposed to oil refinery pollution	*	*	*	*	**	*	*	good
Tanyanont, 2012 [7]	Exposure to volatile organic compounds and health risks among residents in an area affected by a petrochemical complex in Rayong, Thailand	*	*	*		**	*	*	good
Rovira, 2014 [14]	Asthma, respiratory symptoms and lung function in children living near a petrochemical site	*	*		*	**	*	*	good
Chiang, 2016 [30]	Increased incidence of allergic rhinitis, bronchitis and asthma, in children living near a petrochemical complex with SO_2 pollution	*	*	*	*	**	*	*	good

Bustaffa, 2018 [31]	Respiratory symptoms in relation to living near a crude oil first treatment plant in Italy: a cross-sectional study	*	*	*		**	*	*	good

[a] Representativeness of the sample: The studies choose the samples which were truly or somewhat representative of the average in the target population or not. [b] Sample size: The sample size the study selected was justified and satisfactory or not. [c] Non-respondents: If comparability between respondents and non-respondents' characteristics was established, and the response rate was satisfactory, we assigned one star. If the response rate is unsatisfactory, or no description of the response rate, we did not assign star. [d] Ascertainment of the exposure: If the study applied validated measurement tool to ascertain the risk factors, we assigned two stars. Additionally, we still assigned one star to the study applied non-validated measurement tool which was available or described. [e] The subjects in different outcome groups are comparable: The study controlled for the confounding factors or not. [f] Assessment of the outcome: The study applied independent blind assessment, record linkage, self-report or no description. If it used independent blind assessment or record linkage, we assigned two stars. If it used self-report, we assigned one star. g Statistical test: If the statistical test used to analyze the data was clearly described and appropriate, and the measurement of the association was presented, including confidence intervals and the probability level (i.e., p value), we assigned one star. h Quality: The quality of the study; We assigned stars to evaluate study quality, with nine to ten stars indicating "very good" quality, seven to eight stars indicating "good" quality, five to six stars indicating "satisfactory" quality, and zero to four stars indicating "unsatisfactory" quality.
